# An unusual recurrent high-grade glioneuronal tumor with *MAP2K1* mutation and *CDKN2A/B* homozygous deletion

**DOI:** 10.1186/s40478-019-0763-x

**Published:** 2019-07-09

**Authors:** Barry Cheaney, Stephen Bowden, Katie Krause, Emily A. Sloan, Arie Perry, David A. Solomon, Seunggu Jude Han, Matthew D. Wood

**Affiliations:** 1 School of Medicine, Oregon Health & Science University, Portland, Oregon USA; 2 Department of Neurological Surgery, Oregon Health & Science University, Portland, Oregon USA; 3 Department of Pathology, University of California San Francisco, San Francisco, California, USA; 4 Department of Pathology, Oregon Health & Science University, Mail Code L-113, 3181 SW Sam Jackson Park Road, Portland, OR 97202 USA

**Keywords:** Glioneuronal tumor, Anaplastic, *MAP2K1* mutation, *CDKN2A/B* homozygous deletion, Multinodular and vacuolating neuronal tumor of the cerebrum, Next-generation sequencing

## Main text

Ganglioglioma is a WHO grade I glioneuronal tumor composed of neoplastic glial cells and dysmorphic ganglion cells [13]. They account for up to 2% of central nervous system neoplasms, typically occur in the temporal lobes, and are frequently epileptogenic [7]. A majority have alterations that activate the mitogen-activated protein kinase (MAPK) signaling pathway. Following complete resection, recurrence or anaplastic progression has been reported in rare cases [1, 5, 7]. Anaplastic ganglioglioma is a WHO grade III neoplasm with a poor prognosis that may either arise de novo or secondary to the malignant transformation of a previous ganglioglioma [10, 11, 14].

**Fig. 1:**
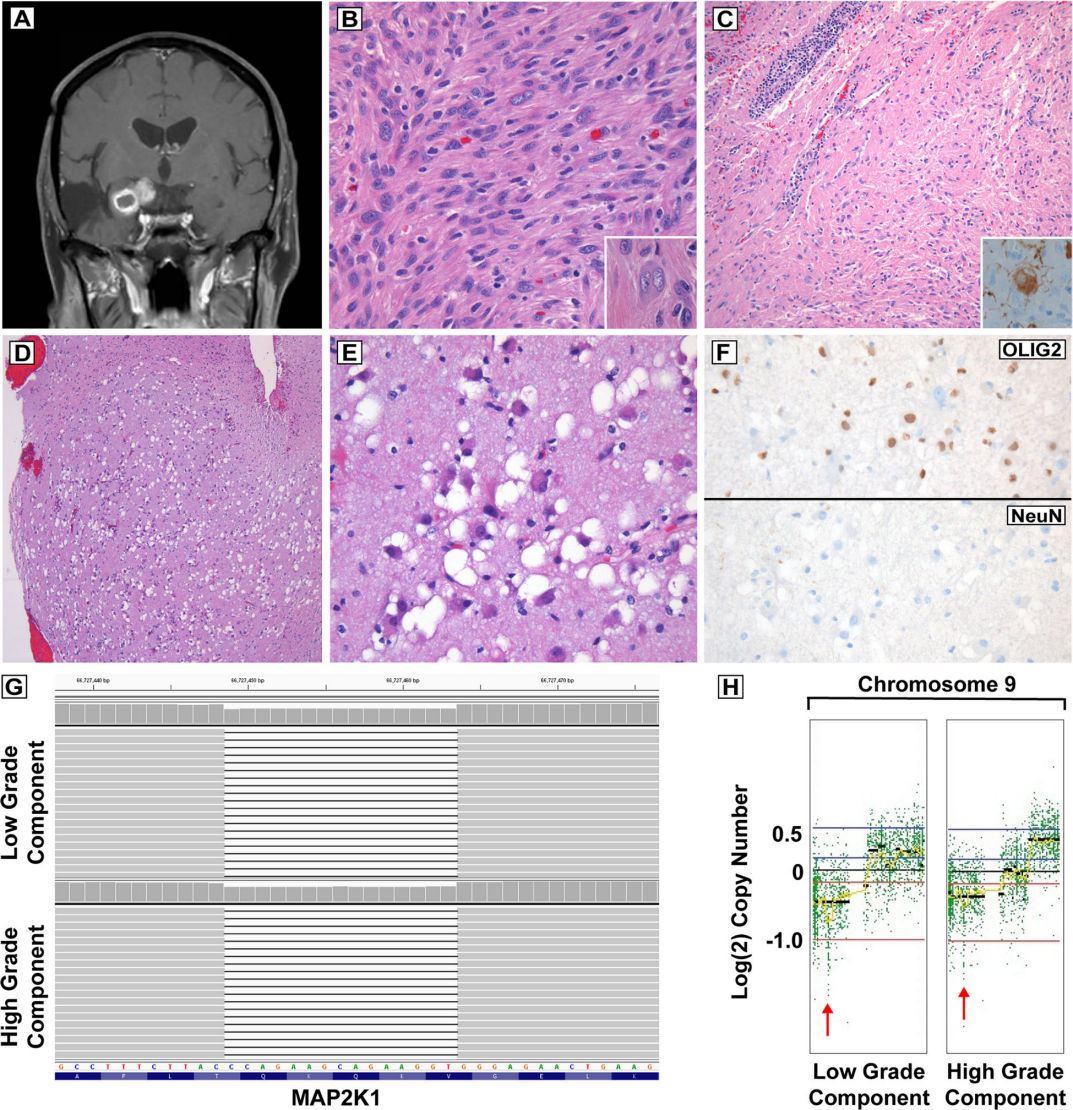
Radiologic, histologic, and molecular features of a recurrent anaplastic glioneuronal tumor in a 71-year-old male. T1-weighted, post-contrast imaging shows a ring-enhancing mass adjacent to the right temporal resection cavity (**a**). Histologic patterns included compact fascicular regions including binucleated ganglion-like cells (**b**, inset), and lower cellularity regions with perivascular lymphocytic inflammation and abnormal ganglion cell body staining for neurofilament (**c**, inset). Still other regions had a nodular pattern with ganglion-like cells showing numerous vacuoles, but containing OLIG2 positive and NeuN negative cells (**d**-**f**). Next-generation sequencing revealed a small in-frame deletion in exon 2 of *MAP2K1* resulting in p.Q56_V60del in both low-grade and high-grade tumor components (**g**). Both components also harbored focal homozygous deletion of the *CDKN2A/B* tumor suppressor genes on chromosome 9p21 (**h**). Full genome-wide copy number profiles are provided in additional file

We present the case of a 71-year-old man with a history of a right temporal mass that was resected and diagnosed as “ganglioneuroma” according to pathology reports from approximately 30 years previous. The patient presented to our institution with recurrent seizures. Neuroimaging revealed an enhancing mesial right temporal lobe mass, invading the right suprasellar and ambient cisterns and the right cerebral peduncle medial to the previous resection cavity. Interval imaging 5 months later showed increasing size and development of ring enhancement (Fig. 1a). He underwent a right pterional craniotomy for tumor debulking. Hematoxylin and eosin stained tumor sections showed that the dominant pattern featured a highly cellular, compact neoplasm with pleomorphic spindled cells with eosinophilic, fibrillary cytoplasm arranged in a sheet-like or fascicular growth pattern, with focal nodules of large cells with bizarre pleomorphic nuclei (Fig. 1b). Some tumor cells showed abundant amphophilic cytoplasm and enlarged nuclei with vesicular chromatin, prominent nucleoli, and occasional binucleation (Fig. 1b, inset). Focal necrosis with peripheral macrophage accumulation was observed. The mitotic index was 5 mitoses per 10 high-power fields. In some regions, the tumor transitioned to lower cellularity with haphazard clusters of dysmorphic ganglion-like cells, eosinophilic granular bodies, and blood vessels with perivascular lymphocytes (Fig. 1c); abnormal cell body positivity for neurofilament protein was also encountered (Figs. 1c, inset). Other low-cellularity regions showed a nodular growth pattern and numerous vacuole-containing ganglion-like cells, which were OLIG2 positive and NeuN negative (Fig. 1d-f). Other immunohistochemical stains showed that the cellular, pleomorphic component was GFAP positive, with scattered cells positive for neuron-specific enolase, and the Ki67 index was approximately 10%. ATRX expression was retained in tumor nuclei in both components, p53 staining was positive in only scattered tumor nuclei, and an IDH1-R132H stain was negative for mutant protein expression. Extravascular CD34 staining was seen in the low-grade ganglioglioma-like component, highlighting occasional cells with ramified branching processes. CD34 staining in the high-grade component only highlighted small vessels, and was negative for extravascular staining. Collagen IV was negative for tumor cell associated staining in all components, highlighting only vessels and a focal fibrotic nodule. The features supported a diagnosis of a high grade glioneuronal tumor, most consistent with anaplastic ganglioglioma, WHO grade III. Prior pathology slides were requested for review, but they had unfortunately been discarded.

Molecular profiling was performed on microdissected regions from low- and high-grade regions of the tumor using the UCSF 500 Cancer Gene Panel, a hybrid capture based next-generation sequencing platform that assesses for mutations and small insertions/deletions in over 500 cancer-associated genes, as well as genome-wide copy number analysis and assessment for common oncogenic rearrangements [3]. Molecular profiling showed an identical small in-frame deletion within exon 2 of the *MAP2K1* gene (p.Q56_V60del) at a mutant allele frequency of approximately 30% in both components (Fig. 1g). Both components also showed a focal homozygous deletion on chromosome 9p21 encompassing the *CDKN2A* and *CDKN2B* tumor suppressor genes (Fig. 1h). Some divergent copy number changes were noted (Additional file 1: Figure S1). The low grade component showed gain of 9q and losses of 9p and 13q, and the high grade component showed gains of 1q, proximal 7q, distal 9q, 15q, 16p, interstitial 16q, and 21q, and losses of distal 7q, 9p, 10, 13q, and portions of 16q. No mutations or rearrangements involving the *BRAF* gene were identified, and alterations typical of diffuse gliomas were not identified (e.g. *IDH1/2* mutation, *EGFR* amplification, *PTEN* mutation/deletion, *TERT* promoter mutation).

The shared alterations of *MAP2K1* mutation and *CDKN2A/B* homozygous deletion supports that low and high grade elements in this tumor represent morphologically distinct components of a single clonal neoplasm. Some lower grade tumor components are consistent with ganglioglioma, while other areas are compatible with multinodular and vacuolating neuronal tumor of the cerebrum (MVNT), an entity first characterized in a series of 10 cases by Huse et al.*,* [2]. MVNT preferentially involves the temporal lobes and commonly presents with seizures. It is clinically indolent, even after incomplete resection, though experience with these tumors is limited to small series [2, 7, 12]. MVNT cells are typically OLIG2 and synaptophysin immunopositive, GFAP and NeuN nonreactive, and are variably associated with ramified CD34 labeling in the adjacent parenchyma [2, 6].

Shared histopathologic features between this case and MVNT include foci of nodular growth and tumor cells with vacuolar alteration and a compatible immunophenotype. There is also genetic overlap between this tumor and MVNT. Pekmezci, et al. recently reported alterations in exon 2 of the *MAP2K1* gene in MVNT, with three small in-frame deletions identified among five of eight MVNT cases [6]. Two cases in that series showed mixed MVNT/ganglioglioma morphology. Interestingly, *MAP2K1* alterations were not identified in a recent study of 40 classic gangliogliomas [7]. At our institution we recently encountered a case of MVNT with the same *MAP2K1* p.Q56_V60del as identified in this report*.* A role for *CDKN2A/B* homozygous deletion in MVNT has not previously been described, but has been observed in anaplastic ganglioglioma and anaplastic pilocytic astrocytomas [1, 9]. The differential diagnosis of pleomorphic xanthoastrocytoma (PXA) was considered, noting that anaplastic PXA is another tumor that often shows *CDKN2A/B* homozygous deletion [8]. The histologic findings and immunophenotype are less compatible with that diagnosis, and there were no *BRAF* or *RAF1* alterations found including *BRAF* p.V600E, *BRAF* fusion, or *RAF1* fusion - alterations which are reported in PXA [4, 8]. To our knowledge, *MAP2K1* exon 2 in-frame deletion has not been reported in PXA to date.

The primary limitation of this report is the lack of pathology slides or tissue from the patient’s original surgery. We cannot determine for certain whether the original tumor was a ganglioglioma, MVNT, mixed ganglioglioma/MVNT, or even another type of low-grade neuroepithelial neoplasm. We cannot evaluate the patient’s original tumor for the genetic alterations that we identified at recurrence. The prior diagnosis of “ganglioneuroma” is unusual and likely reflects evolution in diagnostic terminology over the last few decades; nevertheless, the true nature of the patient’s original tumor is uncertain.

In summary, we report an anaplastic glioneuronal tumor compatible with anaplastic ganglioglioma and/or MVNT, with a confirmed *MAP2K1* exon 2 in-frame deletion and homozygous deletion of *CDKN2A/B*, thus expanding the molecular spectrum of anaplastic glioneuronal tumors. If the patient’s original tumor indeed included a component of MVNT, this would be the first anaplastic example reported to date.

## Additional file

Additional file 1: **Figure S1.** Genome-wide copy number profiles for low-grade (top) and high-grade (bottom) histologic regions of the anaplastic glioneuronal tumor. (TIF 11747 kb)

## Data Availability

All data generated or analysed during this study are included in this published article and its additional files.

## Abbreviations

MAPK: Mitogen activated protein kinase
MVNT: Multinodular and vacuolating neuronal tumor of the cerebrum
PXA: Pleomorphic xanthoastrocytoma
UCSF: University of California San Francisco
WHO: World Health Organization
