# Supplementary figures and images for: An unusual recurrent high-grade glioneuronal tumor with MAP2K1 mutation and CDKN2A/B homozygous deletion
Source: Acta Neuropathol Commun. 2019 Jul 9;7:110. doi: 10.1186/s40478-019-0763-x (PMC6617605; doi:10.1186/s40478-019-0763-x)

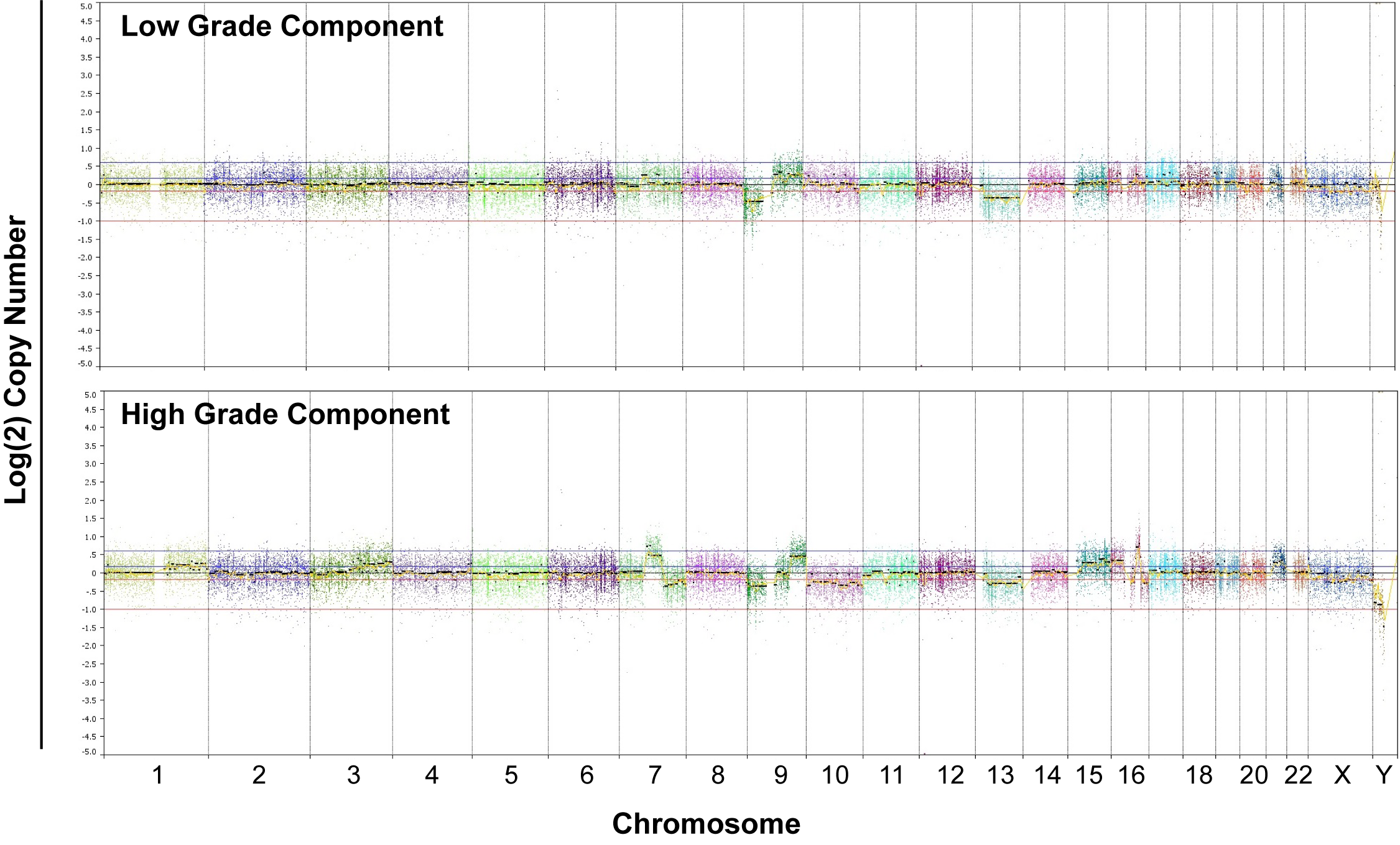

Supplement: Supplementary file 1 — Figure S1. Genome-wide copy number profiles for low-grade (top) and high-grade (bottom) histologic regions of the anaplastic glioneuronal tumor. (TIF 11747 kb) [file 40478_2019_763_MOESM1_ESM.tif]
